# Antiphospholipid Syndrome: Insights into Molecular Mechanisms and Clinical Manifestations

**DOI:** 10.3390/jcm13144191

**Published:** 2024-07-18

**Authors:** Alessandra Ida Celia, Mattia Galli, Silvia Mancuso, Cristiano Alessandri, Giacomo Frati, Sebastiano Sciarretta, Fabrizio Conti

**Affiliations:** 1 Rheumatology, Department of Clinical Internal, Anesthesiological e Cardiovascular Sciences, Sapienza University of Rome, 00161 Rome, Italy; alessandraida.celia@uniroma1.it (A.I.C.); silvia.mancuso@uniroma1.it (S.M.); cristiano.alessandri@uniroma1.it (C.A.); fabrizio.conti@uniroma1.it (F.C.); 2 Department of Medical-Surgical Sciences and Biotechnologies, Sapienza University of Rome, 04100 Latina, Italy; giacomo.frati@uniroma1.it (G.F.); sebastiano.sciarretta@uniroma1.it (S.S.); 3 Maria Cecilia Hospital, GVM Care & Research, 48033 Cotignola, Italy; 4 IRCCS Neuromed, 86077 Pozzilli, Italy

**Keywords:** antiphospholipid antibodies, immunothrombosis, endothelial dysfunction

## Abstract

Antiphospholipid syndrome (APS) is a complex systemic autoimmune disorder characterized by a hypercoagulable state, leading to severe vascular thrombosis and obstetric complications. The 2023 ACR/EULAR guidelines have revolutionized the classification and understanding of APS, introducing broader diagnostic criteria that encompass previously overlooked cardiac, renal, and hematologic manifestations. Despite these advancements, diagnosing APS remains particularly challenging in seronegative patients, where traditional tests fail, yet clinical symptoms persist. Emerging non-criteria antiphospholipid antibodies offer promising new diagnostic and management avenues for these patients. Managing APS involves a strategic balance of cardiovascular risk mitigation and long-term anticoagulation therapy, though the use of direct oral anticoagulants remains contentious due to varying efficacy and safety profiles. This article delves into the intricate pathogenesis of APS, explores the latest classification criteria, and evaluates cutting-edge diagnostic tools and therapeutic strategies.

## 1. Antiphospholipid Syndrome

### 1.1. Definition

Antiphospholipid syndrome (APS) is a systemic autoimmune disorder characterized by a hypercoagulable state which is associated with vascular thrombosis and/or obstetric morbidity, including miscarriage, fetal death, and premature birth [1]. APS is identified by the presence of specific antibodies called antiphospholipid antibodies (aPLs), which include lupus anticoagulant (LA), anti-β-2 glycoprotein 1 (a-β2GP1), and anticardiolipin antibodies (aCL) [1]. Primary APS is diagnosed when there is no clinical or laboratory evidence of another underlying disease, whereas secondary APS is characterized by the presence of another clinical condition such as an autoimmune disorder, infection, medication, or cancer [1,2]. Secondary APS can manifest in association with various conditions including autoimmune diseases such as systemic lupus erythematosus, rheumatoid arthritis, psoriatic arthritis, systemic sclerosis or hemolytic anemia, immune thrombocytopenic purpura, and infectious disease, where aCL and/or LA are frequently observed, potentially leading to clinical manifestations [3,4,5,6].

### 1.2. Classification

The classification of APS, for the identification of homogeneous research cohorts, is currently based on the Sapporo criteria published in 1999 and revised in 2006 [7,8]. The revised Sapporo criteria for APS require clinical features (thrombosis or pregnancy morbidity) and laboratory tests for LAC, IgG/IgM aCL, and/or IgG/IgM anti-β2GPI with at least two aPL tests performed at least 12 weeks apart [7,8]. However, the revised Sapporo criteria did not incorporate certain evidence-based definitions (e.g., microvascular disease or pregnancy morbidity), resulting in the inclusion of a heterogeneous group of “aPL-positive” patients with different risk profiles for research [8].

The 2023 American college of rheumatology (ACR)/European league against rheumatism (EULAR) APS classification criteria expand the scope of manifestations considered indicative of APS [9]. Previously categorized as “non-criteria manifestations”, these now include skin, kidney, heart, and hematologic complications associated with the syndrome. Additionally, the new criteria introduce a stratification method for assessing the risk of macrovascular thrombosis by considering traditional risk factors for venous thromboembolism and cardiovascular disease, with varying diagnostic weights assigned based on the patient’s overall thrombotic risk profile [8]. Furthermore, the 2023 ACR/EULAR criteria incorporate distinct microvascular domain items and redefine pregnancy morbidity, while also adding cardiac valve disease and thrombocytopenia as criteria [8]. The ACR/EULAR criteria showed a good specificity but lower sensitivity. Indeed, when applied to two validation cohorts, the ACR/EULAR criteria achieved a 99% specificity in both cohorts, but showed a sensitivity of 83% in cohort 1 and 84% in cohort 2. In contrast, the Sapporo criteria showed a specificity of 91% in cohort 1 and 86% in cohort 2, with a sensitivity of 100% and 99% in cohorts 1 and 2, respectively [8]. It is essential to recognize that both the modified Sapporo criteria and the 2023 ACR/EULAR criteria serve as classification tools to identify a specific group of patients with APS for clinical studies.

### 1.3. Seronegative Antiphospholipid Syndrome

While these criteria offer a high specificity, they may have a slightly lower sensitivity. Their absence does not rule out an APS diagnosis entirely, but they are commonly used in clinical practice, with clinicians being aware of their limitations and referring complex cases to specialist centers. However, it is not unusual to find patients with clinical manifestations characteristic of APS but with persistently negative aPL tests including aCL and aβ2GPI; IgG and IgM, detected by enzyme linked immunosorbent assay (ELISA); and LA detected by clotting assays according to the guidelines of the International Society on Thrombosis and Haemostasis in daily clinical practice. A diagnosis of seronegative APS (SN-APS) has been suggested for these patients [10,11]. In fact, despite the recommended aPL in routine laboratory practice, aPLs represent a heterogeneous family of antibodies that react with serum phospholipid-binding plasma proteins (mainly β2GPI, prothrombin, protein C, protein S, annexin V, annexin II, and oxidized low-density lipoprotein), phospholipid–protein complexes, and anionic phospholipids [12].

These non-criteria aPLs, along with different methodological approaches, such as “TLC”, has been expanded to thin-layer chromatography (TLC) immunostaining for aPL detection, and could aid physicians in diagnosing and managing patients with SN-APS [13]. Indeed, a recent systematic review comparing non-criteria antiphospholipid syndrome (NC-APS), primarily SN-APS, with definite APS, demonstrated that SN-APS patients have a similar prognosis to those with APS. The systematic review, which included 3798 participants, found no significant differences in the prevalence of thrombosis or pregnancy complications between NC-APS and definite APS. Additionally, in terms of recurrent thrombosis, patients with SN-APS showed comparable outcomes to those with definite APS.

Zohoury and colleagues identified anti-vimentin/cardiolipin (aVim/CL) and anti-phosphatidylserine/prothrombin (aPS/PT) as the non-criteria aPL with the highest sensitivity in SN-APS patients [14]. Additionally, other studies on SN-APS patients revealed associations between non-criteria aPL and clinical manifestations. Specifically, double positivity (aCL by TLC-immunostaining plus aVim/CL by ELISA) exhibited a likelihood positive ratio of 8 for presenting mixed thrombotic and obstetrical features. Furthermore, in SN-APS patients, aCL by TLC-immunostaining correlated with brain magnetic resonance imaging indicating ischemic changes and migraine, while aVim/CL was linked with the presence of livedo reticularis [15] and thrombocytopenia, and anti-carbamylated-β2GPI were associated with thrombocytopenia [16]. Additionally, APS patients testing positive for aVim/CL displayed a higher prevalence of pregnancy morbidity and thrombocytopenia [17].

Therefore, non-criteria aPLs can support the diagnosis in patients with SN-APS, identify clinical subsets, and guide the physician in treatment choices considering that patients with SN-APS seem to have the same prognosis as patients with definite APS.

### 1.4. Clinical Aspects

Thrombotic events are the most common manifestations in patients with APS, which can affect vessels of any size, both venous and/or arterial, and in any body district. Among these, deep vein thrombosis (DVT) is the most common, particularly affecting the lower limbs and often bilaterally. Other less common occurrences include superficial vein thrombosis, sagittal sinus thrombosis, venous circulation impairment in the upper limbs, and vena cava thrombosis. Complications such as pulmonary embolism can arise in approximately one-third of DVT cases. Additionally, arterial thrombosis primarily targets the cerebrovascular system, leading to ischemic strokes. Myocardial infarction and cerebrovascular events are recognized as leading causes of mortality, and myocardial dysfunction in APS can result from coronary artery or microvascular thrombosis [18]. APS patients exhibit an increased risk of premature accelerated atherosclerosis. Despite traditional cardiovascular risk factors being similar to the general population, APS patients have higher instances of carotid and femoral atherosclerotic plaques [18]. 

The most frequent cardiac manifestations during the 10-year follow-up of the total cohort of 1000 APS patients are valve thickening/dysfunction, vegetations, and myocardial infarction [19]. Therefore, valvulopathy was included in the latest classification criteria. The Libman–Sacks endocarditis is characterized by thickening and/or amicrobic valvular vegetations mainly affecting the mitral valve, which will result in insufficiency [9,20]. 

The mitral and aortic valves are most frequently affected with one-third of primary APS (PAPS) patients showing valve disease on transthoracic echocardiography (TTE). The prevalence of HVD in PAPS ranges from 10% to over 60%, and is higher in patients with SAPS. Histopathologically, APS-associated valvulopathy shows fibrosis, calcification, vascular proliferation, and thrombosis. Immunoglobulin and complement deposits are often found on the valve leaflets [18].

Obstetric pathology—recurrent miscarriages, fetal deaths, and preterm births secondary to placental insufficiency—represents the other group of clinical manifestations included in the Sapporo and ACR/EULAR classification criteria of APS [8,9]. 

Due to the efforts of the subcommittee formed to evaluate aPL-nephropathy, this condition was incorporated into the ACR/EULAR classification criteria. Additionally, the subcommittee provided a revised definition for acute and chronic aPL-nephropathy based on histopathological findings (acute: thrombotic microangiopathy; chronic: organized microthrombi or recanalization, fibrous and fibrocellular occlusion, fibrous intimal hyperplasia, and focal cortical atrophy with or without thyroidization) and the presence of aPL [21].

In a small subgroup of patients with APS, there may be a rapid development of multiple thromboses in small-caliber vessels, resulting in multi-organ failure and high mortality, setting up the catastrophic form of APS (Catastrophic Antiphospholipid Syndrome, or CAPS) [22]. Data from the “CAPS Registry”, that included 749 patients accounting for 778 CAPS events, revealed that 404 (52%) had cardiac involvement, mainly heart failure (55%), valvulopathy (31%), and acute myocardial infarction (28%) [23]. Remarkably, 48 out of 58 patients (83%) showed pathological evidence of cardiac thrombotic microangiopathy upon biopsy or autopsy. Additionally, it is noteworthy that cardiac involvement did not correlate with increased mortality [23].

Patients with APS demonstrate accelerated atherosclerosis associated with aPLs [24]. This enhanced atherosclerosis is tied to common internal mechanisms shared between APS thrombo-inflammation and atherogenesis. These mechanisms involve innate immunity dysregulation mediated by aPLs, oxidative stress, and endothelial dysfunction. Furthermore, both processes entail platelet activation and aggregation, thrombin generation, and the simultaneous differentiation of macrophages into foam cells [24,25]. Therefore, it is unsurprising that 33 out 64 (51.6%) APS patients presented a new atherosclerotic plaque at the ultrasound of the carotid and femoral arteries during the 7-year follow-up [26]. Notably, after adjustment for confounders, APS patients had a threefold higher risk of atherosclerosis progression compared to healthy controls (OR = 3.07, *p* = 0.007) and a twofold higher risk than patients with diabetes mellitus (OR = 2.25, *p* = 0.047). A multivariate analysis indicated that plaque progression was independently associated with SLE and traditional cardiovascular risk factors. Additionally, achieving sustained low-density lipoprotein target levels reduced the risk of plaque progression [26].

## 2. Pathogenesis

### 2.1. Endothelial Dysfunction

The endothelium functions as a crucial regulator of vascular homeostasis, equipped with mechanisms that counteract thrombosis and inflammation. Heparinoid proteoglycans, prostacyclins, protein C receptor, and tissue factor pathway inhibitor collectively contribute to maintaining an antithrombotic endothelial surface [27]. Endothelial dysfunction is a change in the normal endothelial function that involves the loss of structural and functional characteristics contributing to the increased cardiovascular risk. Endothelial dysfunction is the result of multiple pathological changes including the endothelium activation, a condition mainly defined by the expression of adhesion molecules [28]. It is typically induced by pro-inflammatory cytokines, which facilitate the recruiting and adhesion of circulating leukocytes to the vessel wall [28]. In the presence of inflammatory cytokines such as interleukin-1 (IL-1), IL-6, and the soluble interleukin 2 receptor (sIL2-R), endothelial cells undergo a transition and participate in hosting defense by inducing the expression of genes, recruiting cells from the autoimmune system, and increasing permeability and thrombotic potential. In APS, aPL can directly target endothelial cells, leading to endothelial dysfunction and activation. Mechanistically, aPL engages surface receptors on endothelial cells, triggering signaling pathways associated with inflammation and thrombosis. In animal models, aPL administration increases tissue factor activity and promotes leukocyte-endothelium interactions, contributing to thrombotic events [29]. The binding of anti-β2-glycoprotein 1 antibodies to β2-glycoprotein 1 at the cell surface results in the activation of cultured endothelial cells, platelets, monocytes, neutrophils, fibroblasts, and trophoblasts, as well as the expression and release of cell-type-dependent activation markers. Animal models have confirmed that the infusion of anti-β2-glycoprotein 1 antibodies increases the protein expression of tissue factor, which is responsible for the activation of the coagulation cascade, in monocytes and vascular homogenates [30]. Moreover, in vitro studies demonstrate that aPLs directly activate endothelial cells, inducing the expression of adhesion molecules and tissue factor, and endothelium-derived microparticles detected in APS patients’ circulation indicate endothelial activation and suggest a predisposition for leukocyte-endothelium interactions [31,32]. 

However, in APS, the normally quiescent endothelium undergoes activation, shedding its antithrombotic profile and assuming a proinflammatory phenotype [33]. This transition facilitates interactions between leukocytes and endothelial cells, leading to leukocyte extravasation and vascular inflammation. The disruption of key molecules involved in leukocyte–endothelium interactions can mitigate aPL-mediated thrombosis, highlighting the significance of endothelial dysfunction in APS pathophysiology. Additionally, endothelial dysfunction in APS extends beyond thrombosis, contributing to the pathogenesis of other clinical manifestations such as vascular inflammation, atherosclerosis, and organ damage. Understanding the intricate interplay between aPL and endothelial cells is essential for elucidating the pathophysiology of APS and developing targeted therapeutic strategies.

### 2.2. Thrombosis

Injecting aPLs into mice, rats, or hamsters does not induce spontaneous thrombotic complications. However, in accordance with the ‘multi-hit’ hypothesis of thrombosis, the thrombotic reaction following a priming event, like a minor vascular injury, is notably intensified when aPLs are present compared to the infusion of a control antibody [34]. This observation in animal models fits with the finding that aPLs are risk factors for thrombosis in humans. Animal models have clearly shown that antibodies against β2-glycoprotein 1, especially those against domain 1, can induce a strong prothrombotic phenotype [35,36]. According to one study, aCLs have the potential to elevate the risk of thrombosis in mice, regardless of β2-glycoprotein 1 and prothrombin [37].

### 2.3. Complement

Patients with primary APS had lower serum levels of C3 and C4 compared to healthy donors and those with non-lupus connective tissue diseases. Significant inverse correlations were observed between the C3 or C4 levels, and increased levels of C3a or C4a in the sera of primary APS patients. This suggests that the hypocomplementemia in primary APS is due to the consumption of complement proteins and the activation of the complement pathway [38]. Complement proteins are involved in the development of aPL-related thrombosis. An activated complement system has been linked to a prothrombotic state through the membrane attack complex or anaphylatoxins, especially C5a. Anaphylatoxins activate monocytes or endothelial cells via specific receptors on their surfaces, leading to the production of prothrombotic molecules such as tissue factors. Recently, it has been reported that the interaction between the complement system and platelets may also contribute to complement-related thrombogenicity [38].

## 3. Therapeutic Aspects

Thromboprophylaxis is a significant challenge in the management of APS. Primary thromboprophylaxis refers to the prevention of thrombosis in individuals with no history of clots, while secondary thromboprophylaxis aims to prevent the recurrence of clots after an initial thrombotic event. The key to primary thromboprophylaxis is the conventional management of cardiovascular risk factors through lifestyle changes.

The management of asymptomatic individuals with persistent aPLs is tailored based on each person’s circumstances, particularly considering any additional cardiovascular risk factors. For those with a high-risk profile, characterized by high antiphospholipid antibody titers, triple positivity, other cardiovascular risk factors, or other systemic autoimmune diseases, primary prevention with low-dose aspirin (LDA) might be recommended. The use of LDA for primary prophylaxis is backed by a meta-analysis of seven observational studies involving 460 asymptomatic antiphospholipid antibody carriers. This analysis found that the risk of a first thrombosis was reduced by half in those who took LDA compared to those who did not [39]. It should be clarified that the protective effect of LDA was evidenced in the prevention of arterial thrombosis and not venous thrombosis. Notably, this effect was observed mainly in retrospective studies rather than prospective trials [39]. Hydroxychloroquine has been utilized in clinical practice based on empirical evidence and in vitro studies, although no rigorous RCTs have been conducted to date [40,41]. Unprovoked venous thrombosis and arterial thrombosis are of concern and should be treated with indefinite anticoagulation therapy with a vitamin K antagonist (VKAs, such as warfarin) or, occasionally, low-molecular weight heparin. Data from a RCT focusing solely on patients experiencing venous events, along with combined data from five studies predominantly involving venous events, indicated that there was no added advantage of aiming for a target international normalized ratio (INR) of 3–4 compared to an INR target of 2–3 [42,43,44,45]. Despite the increasing utilization of direct oral anticoagulants (DOACs) for the secondary prevention of thrombosis in the general population, there is a dearth of evidence regarding their efficacy and safety in APS. A retrospective analysis of APS patients enrolled in three RCTs comparing dabigatran to warfarin and in one RCT comparing rivaroxaban to warfarin for venous thrombotic APS revealed no disparities in outcomes between DOACs and VKAs [46]. However, this evidence is constrained by small sample sizes, the insufficient representation of high-risk APS patients, and a limited follow-up duration. The first RCT investigating rivaroxaban versus warfarin in APS patients with triple antiphospholipid antibody positivity was halted prematurely due to a heightened incidence of thromboembolic events, mainly arterial, in the rivaroxaban arm [47]. Consequently, rivaroxaban is not recommended for use in APS patients with triple aPL positivity. In three RCTs comparing rivaroxaban to VKAs in APS, rivaroxaban was associated with a higher risk of thrombotic events despite a similar or superior safety profile compared to VKAs [48,49,50]. Methodological weaknesses were evident in these trials, including the premature termination of the TRAPS trial due to arterial thrombosis in rivaroxaban recipients and the failure to meet non-inferiority thresholds in the study by Ordi-Ros et al. [48]. Additionally, the recent ASTRO-APS trial evaluating apixaban versus VKA was halted prematurely, with disappointing results, showing more thrombotic events in the apixaban group [50]. Collectively, RCTs comparing DOACs with VKAs present significant limitations in their design and suffer from small sample sizes, leading to insufficient statistical power. This issue is exacerbated by the challenge of conducting adequately powered RCTs, particularly when dealing with a rare medical condition suggesting how observational studies may offer some remedy to this shortfall, albeit only partially, by providing additional insights into the effectiveness and safety of DOACs in APS patients [51]. The recent systematic review and meta-analyses of RCTs found that DOACs seem to be less effective than VKA for the prevention of thrombotic events, particularly of arterial thrombotic events [52,53].

In this scenario, the consensus among experts is that DOACs could be contemplated in patients who struggle to achieve a target INR of 2–3 despite adhering to VKA therapy or in those with contraindications to VKAs, especially if they present single or double positive aPLs without the detection of LA or have a history of prior venous events [54,55]. 

As for CAPS, a form burdened by high mortality and morbidity, the EULAR recommendations suggest a combination therapy of glucocorticoids, heparin, and plasma exchange or IVIG as a first-line approach versus single-agent treatments. For refractory cases of CAPS, B-cell depletion therapies (e.g., rituximab) or complement inhibition therapies (e.g., eculizumab) may be considered, based on the results of some case reports [56].

## 4. Conclusions

The revised classification criteria, including the 2023 ACR/EULAR guidelines, have expanded the understanding and identification of APS, incorporating a broader spectrum of clinical manifestations and risk stratification methods. Despite advancements, the diagnosis of APS remains challenging, particularly in cases of seronegative APS, where traditional aPL tests are negative, yet clinical symptoms persist. Non-criteria aPLs have emerged as valuable tools in diagnosing and managing these patients.

Thrombotic events, particularly deep vein thrombosis, are common in APS, and the inclusion of cardiac manifestations in the latest criteria underscores the syndrome’s systemic impact. Catastrophic APS is a severe form with high mortality, further complicated by accelerated atherosclerosis.

Thromboprophylaxis focuses on managing cardiovascular risk factors with low-dose aspirin and hydroxychloroquine for primary prevention, and long-term anticoagulation with vitamin K antagonists for secondary prevention. The use of direct oral anticoagulants is controversial due to inconsistent evidence and safety concerns, particularly in high-risk patients.

In summary, APS is a complex disorder requiring a nuanced diagnostic and management approach. Ongoing research into non-criteria aPLs and new therapeutic strategies is essential for improving patient outcomes. The evolving criteria and deeper understanding of APS provide a foundation for better clinical interventions and personalized care.
